# Power Enhancement of 265 nm DUV-LED Flip-Chip by HVPE-AlN High-Temperature Annealing

**DOI:** 10.3390/mi14020467

**Published:** 2023-02-17

**Authors:** Wenkai Yue, Ruixuan Liu, Peixian Li, Xiaowei Zhou, Yang Liu, Bo Yang, Yingxiao Liu, Xiaowei Wang

**Affiliations:** 1 School of Advanced Materials and Nanotechnology, Xidian University, Xi’an 710071, China; 2 Shenzhen Zhenhua Microelectronics Co., Ltd., Shenzhen 518000, China; 3 School of Economics and Management, Xidian University, Xi’an 710071, China; 4 State Key Discipline Laboratory of Wide Band Gap Semiconductor Technology, Xidian University, Xi’an 710071, China

**Keywords:** hydride vapor phase epitaxy, high-temperature annealing, aluminum nitride, flip-chip, DUV-LED

## Abstract

In this paper, the X-ray diffraction full width at half the maximum (XRD FWHM) of a 3.5 µm-thick hydride vapor phase epitaxy-aluminum nitride (HVPE-AlN) (002) face after high-temperature annealing was reduced to 129 arcsec. The tensile strain in the HVPE-AlN samples gradually released with the increasing annealing temperature. When the annealing temperature exceeded 1700 °C, an aluminum oxynitride (AlON) region was generated at the contact interface between HVPE-AlN and sapphire, and the AlON structure was observed to conform to the characteristics of Al5O6N by high-resolution transmission electron microscopy (HRTEM). A 265 nm light-emitting diode (LED) based on an HVPE-AlN template annealed at 1700 °C achieved a light output power (LOP) of 4.48 mW at 50 mA, which was approximately 57% greater than that of the original sample.

## 1. Introduction

Aluminum nitride (AlN) thin films have attracted a great amount of attention due to their large bandgap, high thermal conductivity, and high acoustic velocity at high temperatures [1,2,3]. AlN-based electronics hold great promise, particularly in the fields of deep ultraviolet light-emitting diodes (DUV-LEDs) and photodetectors for solar-blind technologies [4,5,6]. DUV-LEDs with high efficiency and reliability may be grown on bulk AlN single crystal substrates with a low dislocation density and sufficient size [7,8]. Presently, the most prominent and successful method of producing large, bulk AlN single crystals is the PVT method, also known as the sublimation-recondensation method, which involves no toxic gases in the process. While this is the best approach for synthesizing bulk AlN single crystal substrates, its high initial cost and profound ultraviolet light absorption limit its use [9,10,11].

AlN films epitaxially grown on heteroepitaxial substrates are fabricating AlN-based devices to further expand their applicability in various situations [12,13]. For example, Miyake et al. [14] developed a high-temperature (HT) annealing approach for AlN that has garnered considerable attention due to its efficacy in enhancing the crystal perfection of sputtered AlN. Additionally, this method considerably reduces the strain in sputtered AlN films [15]. HT-annealed AlN provides a feasible method for fabricating highly efficient UVC-LEDs [16].Using hydride vapor phase epitaxy (HVPE-AlN), a low-cost and DUV transparent AlN/sapphire template with a smooth surface was recently developed, paving the way for the development of homoepitaxy of AlN and Al-rich AlGaN. Although AlN homoepitaxy based on HVPE-AlN has been examined previously, HT annealing based on thicker HVPE-AlN has remained unexplored until now [17,18,19].

Although the research field of UV-LED has made many breakthroughs [20,21,22], the light output power (LOP) of flip-chip DUV-LEDs on sapphire substrates is still in a low development state. Because standard LED contact layer materials are extremely absorbent in the DUV range for AlGaN-based DUV-LEDs, flip-chip designs are typically used [23]. To reduce the light absorption by the n and p contact layers on the epitaxial surface, the DUV light generated by the active layer is extracted through a clear substrate such as sapphire. Due to the obvious total internal reflection (TIR) at the AlN/sapphire surface and the internal optical absorption of the p-AlGaN layer, flip-chip DUV-LEDs on sapphire substrates have poor light extraction and output power.

This paper discusses research where HVPE-AlN samples were subjected to HT annealing (HTA) at different temperatures, and the surface morphology and crystal quality of the samples were compared and analyzed. Additionally, Raman spectroscopy and a combination of X-ray diffraction and response surface methodology (XRD-RSM) were used to analyze stress-strain states of films in HVPE-AlN samples after HTA. Atomic force microscopy (AFM) methods were used to analyze the different AlN surfaces after HT annealing. Furthermore, high-resolution transmission electron microscopy (HRTEM) was used to analyze the aluminum oxynitride (AlON) structure at the AlN/sapphire interface after HT annealing and the AlON generation mechanism. Finally, we fabricated a 265 nm LED flip-chip on a hydride vapor phase epitaxy-aluminum nitride (HVPE-AlN) sample (annealed at 1700 °C) and compared the electrical and luminescent properties of the chip.

## 2. Experimental Method

The substrate was a 2-in, 3.5-μm thick HVPE-AlN (0001) film on a planar sapphire substrate grown by NANOWIN Co., Ltd., Suzhou, China. The sapphire substrate was heated to 1000 °C, NH_3_ was introduced, and then the sample was annealed in a mixed atmosphere of H_2_ under a total pressure of 20 hPa for 10 min. The reactor chamber was maintained at a pressure of 10 hPa during the AlN growth process. The AlCl_3_ and N_2_ flows were 45 and 195 sccm, respectively, and the carrier H_2_ flow was 1950 sccm. AlCl_3_ was produced in the reactor (source zone) by the reaction of Al metal with HCl gas at a temperature of 500 °C. During the growth process, the HCl flux was set to 0.02 sccm, the N_2_ flux was set to 0.5 sccm, and the V/III ratio was kept at 150.

Following growth, the AlN samples were removed from the HVPE system and overlapped face-to-face to prevent the thermal disintegration of the AlN films during HTA. The samples were then covered with graphite plates on both sides to ensure consistent heat conduction and annealed for 3 h at preset temperatures of 1500 °C, 1600 °C, and 1700 °C in a high-temperature annealing furnace. Protective N_2_ gas was employed during the annealing process, with the pressure of the gas set at 0.55 atmospheres and a flow rate of 0.3 L/min. The heteroepitaxial structure for the 265 nm LEDs was grown on the HVPE-AlN substrate using a vertical metalorganic chemical vapor deposition (MOCVD) system (TES HESTIA 2G) that was specifically designed for the production of deep UV LED. Trimethyl aluminum (TMAl), trimethyl gallium (TMGa), silane, bis-cyclopentadienyl magnesium (Cp_2_Mg), and NH_3_ were used as MOCVD precursor materials.

The strain and composition profiles of the samples were analyzed using XRD-RSM. The X-ray diffractometer was the Rigaku SmartLab 9kW. A Bruker Dimension Edge instrument was used for AFM to examine the surface morphology of the AlN samples. The structural quality of AlN was characterized by high-resolution X-ray diffraction (HRXRD). In addition, to study the luminous uniformity of 265 nm LED chips, we characterized the samples under different working currents through a microscopic optical distribution test system.

## 3. Results and Discussion

### 3.1. AFM and XRD Study after HTA of HVPE-AlN

**Figure 1:**
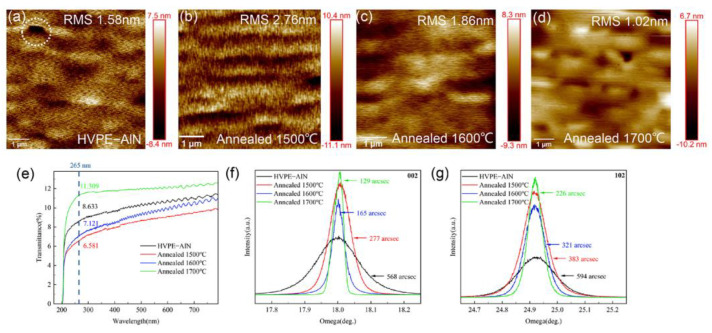
(**a**) HVPE−AlN samples; (**b**) AlN samples annealed at 1500 °C; (**c**) AlN samples annealed at 1600 °C; (**d**) AlN samples annealed at 1700 °C; (**e**) UV–vis plots of four groups of samples; (**f**) (002)-plane XRC plots of four groups of samples; (**g**) (102)-plane XRC plots of four groups of samples.

To investigate the effect of annealing temperatures on the HVPE-AlN template, 3.5-μm thick HVPE-AlN films were annealed at different temperatures. After annealing, all samples showed crack-free upper surfaces. Figure 1a shows the AFM scans of tiny step-like structures unannealed HVPE-AlN with high density covering the entire AlN surface. There were some pore structures in the middle (inside white-dotted line), indicating that the AlN film prepared by the HVPE process had a high degree of uniform deposition. The root means square (RMS) value first increased and then decreased as the annealing temperature increased from 1500 °C to 1700 °C in Figure 1b–d. The reason for the larger RMS value after thermal annealing at 1500 °C shown in Figure 1b may be because face-to-face annealing in a N_2_ environment at 1500 °C did not satisfy the Al, N, and reconstruction requirements of the AlN surface. The solid-phase equilibrium of O resulted in the incomplete reconstruction of the AlN surface atoms. After thermal annealing at 1700 °C, the incomplete reconstruction of the AlN surface atoms tiny step-like structures coalesced to form a smooth platform-like surface morphology, as shown in Figure 1d. Before the full structure of the 265 nm LED was grown by metal-organic vapor phase epitaxy (MOVPE), the crystalline quality differences of HVPE-AlN templates at different temperatures were investigated by XRD. Figure 1f,g shows the dependence of the X-ray rocking curve full width at the half maximum (XRC-FWHM) value of the AlN film on the annealing temperature. After annealing at 1600–1700 °C, the FWHMs of the (002)-plane XRC of the AlN films decreased to the range of 129–165 arcsec. Meanwhile, the FWHM of the (102)-plane XRC after annealing at 1500 °C, 1600 °C, and 1700 °C decreased from 594 to 383, and 321 and 226 arscec, respectively. The sharp decrease in (102)-plane XRC-FWHMs with the increasing annealing temperature implies that the grain size increased due to the elimination of twist components and domain boundaries. With increasing annealing temperature, the XRC-FWHM values of the four groups of samples gradually decreased and the amplitude gradually decreased. In all four AlN templates, edge dislocations dominated. In addition, the large difference in the edge dislocation and screw dislocation of these four AlN templates may have been one of the main reasons for the different surface morphologies.

### 3.2. UV−Vis Study after HTA of HVPE−AlN

Defects significantly impact not only the electrical characteristics of AlN-based optoelectronic devices but also the AlN optical properties. Figure 1e shows the UV light transmittance spectra of the AlN epitaxial layers deducted from the air background before and after high-temperature annealing. The transmittances of the four groups of samples are 8.633, 6.581, 7.121, and 11.309. The clear Fabry-Pérot interference fringes in the spectrum indicate a distinct interface between the AlN film and the sapphire. The transmittance of the HVPE-AlN sample after annealing at 1700 °C was the highest among the four samples, indicating the lowest number of defects for light scattering and absorption. It has been proven by relevant research [24] that the transmittance of HTA-AlN is higher than the unannealed samples, suggesting that the amount of the defects causing light scattering and absorption is reduced after thermal treatment, in accordance with the variation in extended defect densities (revealed by HRXRD). The transmittance results in this manuscript are consistent with the XRD results.

### 3.3. Volcano-like Protrusions Visible on the 1700 °C HTA-AlN Surface

**Figure 2:**
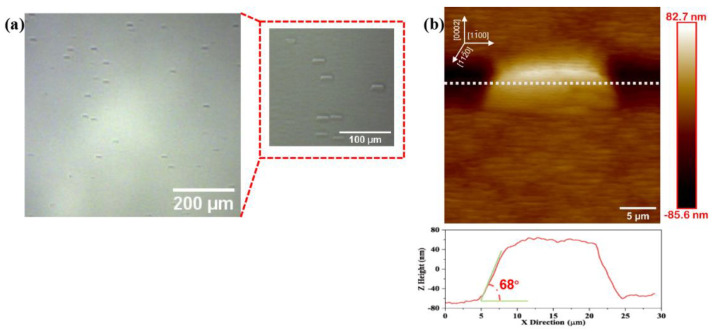
(**a**) Optical microscope image of the surface of an AlN sample annealed at 1700 °C; (**b**) AFM image of volcano-like protrusions on the surface of an AlN sample annealed at 1700 °C.

As shown in Figure 2a, the surface morphology changed significantly after HTA, with volcano-like protrusions visible on the surface with stepped edges. All volcano-like protuberances exhibited similar geometries with a side dip angle of approximately 68°. The lateral edges of the volcano-like protuberance may have been composed of (1014) planes, possibly from the (1014) stacking fault slip plane. Due to weaker atomic bonds, it was easier for atoms near the slip plane to decompose at a high temperature. Since the lateral dimensions of the volcano-like protrusion structures in the HTA-treated AlN were significantly larger than those of the streaks in the as-grown samples, there was some lateral merging between the streaks during the HTA treatment. The FWHMs of the (1012) -plane XRCs decreasing with increasing annealing temperature is the primary factor that contributes to the AlN films lateral merging phenomenon. During the annealing, the AlN films coalesced, resulting in the annihilation of domain boundaries, thus improving the crystallinity of the AlN films [24].

### 3.4. Raman Study after HTA of HVPE-AlN

To deeply study the changes in the HVPE-AlN after annealing at 1700 °C correlate before annealing, we examined the sample by Raman spectroscopy and HRTEM.

**Figure 3:**
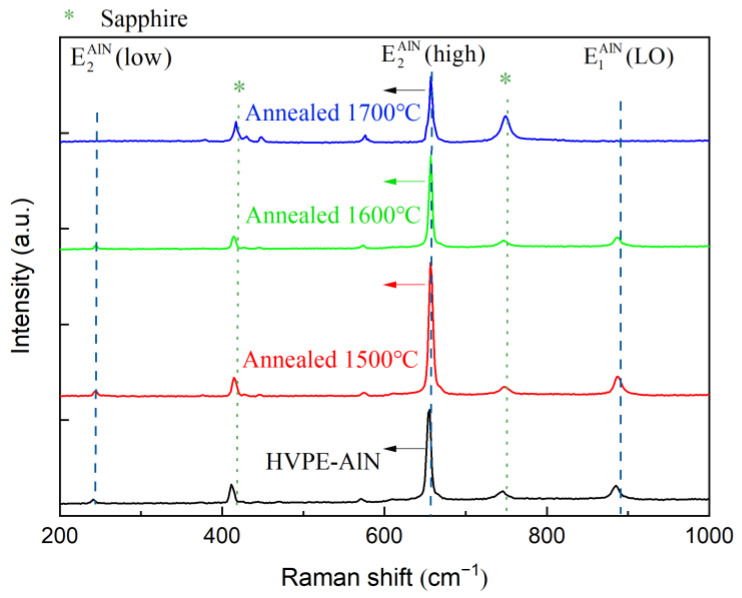
Raman spectra of four groups of AlN samples.

Figure 3 shows that the vertical dashed line represents the unstrained position of AlN-E_2_(high) with a wavenumber of 657.4 cm^−1^. Therefore, the AlN-E_2_(high) Raman peaks of the four samples were blue-shifted relative to the Raman peaks of the unstrained AlN, which indicates the presence of tensile strain in the AlN layers of the four samples. Since the AlN-E_2_(high) mode was polarized parallel to the sample surface, it should have been more sensitive to in-plane strain in the AlN layer [25]. There is a biaxial strain field in AlN. Therefore, the measured displacement of AlN-E_2_(high) relative to AlN*-E_2_(high) in the unstrained state can be used to calculate the respective biaxial in-plane strain *σ*_xx_ in the AlN sample:

(1) $$\sigma_{\mathrm{xx}}=\frac{\Delta \omega_{E_{2}}\left(\mathrm{high}\right)}{k}$$

In the equation, $\Delta \omega_{E_{2}}\left(\mathrm{high}\right)$ is the difference between AlN-E_2_(high) of the tested AlN sample and AlN*-E_2_(high) in the unstrained state. K is the biaxial stress coefficient; here, we took the value of k as 2.55 cm^−1^/Gpa [26]. ‘*’ is the Raman peak of sapphire substrate. The in-plane strain calculation results for four samples are shown in Table 1. The calculation results show that the tensile stress before high-temperature annealing was 0.82 Gpa, the value of the tensile strain gradually decreased with the increasing annealing temperature, and the tensile strain after HTA at 1700 °C was only 0.13 GPa.

### 3.5. HRTEM Study on the 1700 °C HTA-AlN AlN/Sapphire Interface

**Figure 4:**
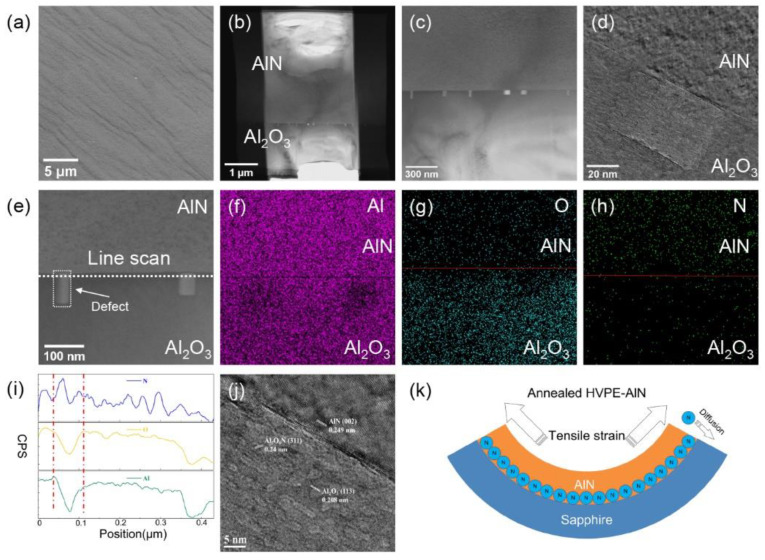
(**a**) SEM image of the surface of an AlN sample annealed at 1700 °C under a magnification of 8000 times; (**b**) STEM image of a cross section under a magnification of 35,000 times; (**c**) STEM image of defects at the AlN/sapphire interface; (**d**) HRTEM image of a single rectangular defect; (**e**) TEM-EDS surface scan at the defect; (**f**) EDS result of Al; (**g**) EDS result of O; (**h**) EDS result of N; (**i**) EDS line scan; (**j**) single defect schematic diagram of element calibration; (**k**) schematic diagram of N diffusion.

Figure 4a shows a top-view SEM image at 8000× magnification of the thin film surface of the AlN sample annealed at 1700 °C. There were still macrosteps generated during the growth process on the surface of the sample and the macrosteps merged in some areas after HTA, which is consistent with our AFM observations. The rectangular white area in the STEM image of the cross-section and the defect in Figure 4b,c is the thermal defect generated after HTA. The distance between adjacent defects was roughly distributed in the range of 120–350 nm. To further characterize the changes inside the defect, HRTEM was used to magnify and observe a single defect. Figure 4d shows that the rectangular defect of the sample was significantly different from the AlN and sapphire structural arrangements. Then, we performed surface scans and line scans of Al, O, and N near the sample defects by transmission electron microscopy-energy-dispersive X-ray spectroscopy (TEM-EDS). The dashed line in Figure 4e represents the direction of the TEM-EDS line scan. Figure 4f–h shows the EDS surface scan results of Al, O, and N, respectively. In the results in Figure 4f,g, we observed that the Al and O densities decreased at the rectangular defect sites, which was consistent with the line scan results in Figure 4i. However, Figure 4f clearly shows that the N distribution appears on the side of the sapphire substrate. Combined with the HRTEM calibration results in Figure 4j, the HRTEM image shows a clear boundary between the AlN layer and the Al_2_O_3_ layer. Specifically, the lattice spacings of the different regions were measured as 0.249 nm and 0.208 nm, which were well indexed to the (002) facet of AlN (PDF# 25-1133) and the (113) face of Al_2_O_3_ (PDF# 10-0173), respectively. In addition, the region between the AlN layer and the Al_2_O_3_ layer showed new lattice fringes distinct from those of AlN and Al_2_O_3_ with a lattice spacing of 0.24 nm, possibly ascribed to the (311) plane of Al_5_O_6_N (PDF# 48-0686). The presence of Al_5_O_6_N was attributed to the diffusion of N2 from the AlN layer to the Al_2_O_3_ layer at temperatures above 1700 °C.

When AlN was annealed in a N_2_ atmosphere at 1700 °C, there was a stark difference from the observations of Fukuyama et al. [27] of some nano-thin epitaxial γ-AlON layers at the AlN/sapphire contact. The appearance of the defect regions of AlON in our layer (Figure 4b) indicates that the AlON formation rate was not uniform and that diffusion was restricted, possibly due to partial regional stress state differences. For mixtures of AlN and α-Al_2_O_3_, this observation is in good agreement with that of Bandyopadhyay et al. [28], where the amount of γ-AlON in the solid-phase combination significantly increased at T > 1670 °C. O and N must diffuse through the lattice for AlON production to occur. With regards to AlN, the O concentration must be greater than its maximal solubility in AlN ${(>2\times 10}^{21}{\mathrm{cm}}^{-3})$ to result in AlON production [29]. Considering that diffusion is a temperature-dependent process, AlON production is more noticeable in samples that have been annealed at higher temperatures. Combined with Figure 4k, we made a diagram of the mechanism of AlON generation. With the increasing HTA temperature, the tensile strain in the AlN epitaxial layer decreases. After a certain thermal diffusion energy barrier is exceeded, O atoms in the Al_2_O_3_ layer are removed from the crystal. Their detachment from the lattice leaves oxygen vacancies, and N atoms diffuse to Al_2_O_3_ through the lattice, forming stable Al_5_O_6_N after the cooling process. After the HTA of the HVPE-AlN thick film, the formed Al_5_O_6_N was enriched in a certain area to form the defect with the rectangular cross-section visible in our HRTEM image. The refractive index of the Al_5_O_6_N rectangular defect was different from that of the surrounding sapphire, which may have reduced the total in-plane reflection of the light at the AlN/sapphire interface, thereby improving the light transmittance of the AlN sample annealed at 1700 °C at 265 nm. 

### 3.6. XRD-RSM Study 

**Figure 5:**
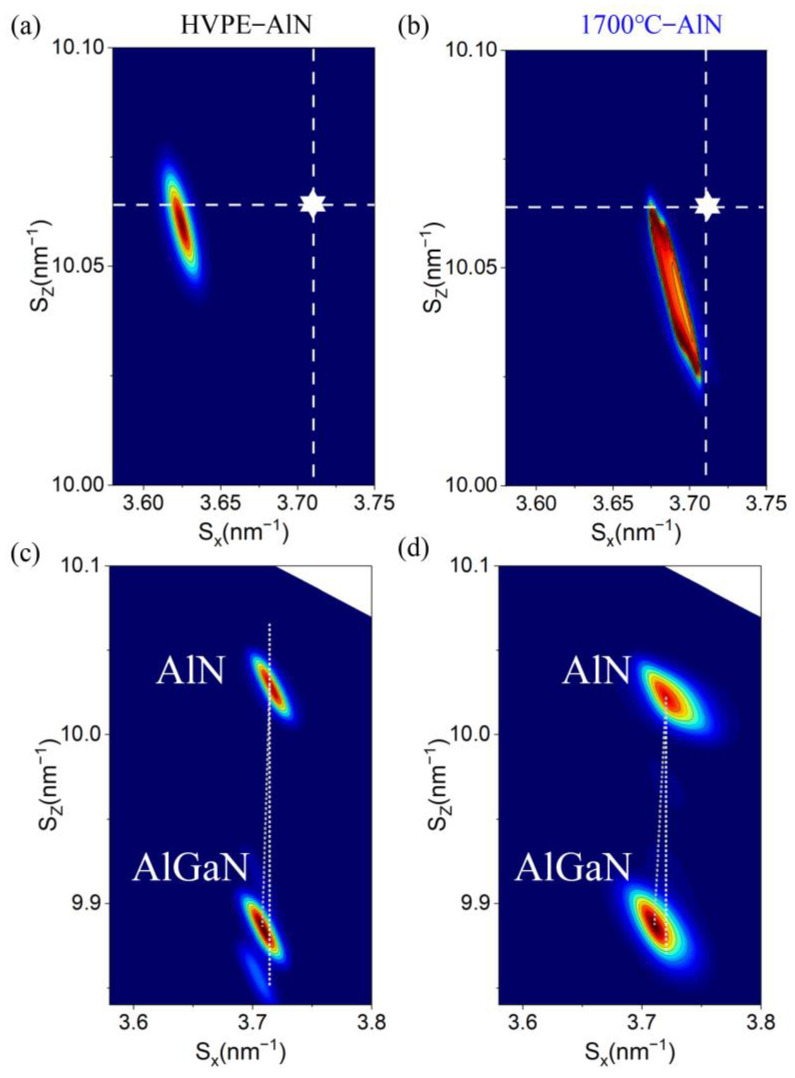
XRD-RSM results of AlN samples before and after high-temperature annealing: (**a**) the unannealed HVPE-AlN sample; (**b**) the high-temperature annealed sample at 1700 °C; (**c**) AlGaN on the unannealed HVPE-AlN sample; (**d**) AlGaN on the high-temperature annealed sample at 1700 °C.

The (105) plane XRD reciprocal space diffraction pattern of the AlN and AlGaN sample is shown in Figure 5. The thickness of the AlGaN layer in the DUV-LED sample is preset to 3.5 μm and the preset Al composition is 75%. Figure 5a shows the HVPE-AlN sample, and Figure 5b shows the AlN sample annealed at 1700 °C. The *S*_*x*_ and *S*_*z*_ values of AlN under unstrained conditions in the X-ray reciprocal space diffraction (XRD-RSM) pattern were 0.2858 and 0.7730, respectively, and the position is marked as a white star in the figure.

According to the RSM diagram, the lattice constant of the sample was determined by the following method [30]:

(2) $$\{\begin{array}{c} a=2\sqrt{\frac{h^{2}+h\cdot k+k^{2}}{3S_{x}^{2}}}\\ c=\frac{l}{S_{z}} \end{array}$$

where (*S*_*x*_, *S*_*z*_) is the reciprocal lattice point (RLP) at the center of the (*hkl*) plane. The coordinates of the center positions of the AlN RLP of HVPE-AlN and AlN annealed at 1700 °C were (3.6251, 10.0597) and (3.6752, 10.0623), respectively. According to Equation (2) and the asymmetric (105)-plane response surface methodology (RSM) results, the AlN lattice constants a and c of the two samples were determined. The lattice constants of the two groups of samples a and c were larger than those of the unstrained state, which indicated that the HVPE samples had obvious tensile strain before and after annealing. The lattice constant c of the AlN sample after HTA was close to the AlN lattice constant C_0_ in the unstrained state, so the tensile strain in the sample after HTA was obviously reduced. This further showed that our Raman spectroscopy results were basically accurate.

Samples HVPE-AlN and 1700 °C annealed HVPE-AlN had AlGaN RLP center position coordinates of (3.7082, 9.8861) and (3.7105, 9.8873) in Figure 5c,d, respectively. The AlGaN lattice constants a and c of samples HVPE-AlN and HVPE-AlN annealed at 1700 °C were determined using Equation (2) and the asymmetry (105)-plane RSM results.

Due to the hexagonal configuration of the III-nitrides, we concluded that they satisfied the biaxial stress requirement. As a result, the lattice constants of the AlGaN layer obeyed the following Equation (3) [31]:

(3) $$\frac{c\left(x\right)-c_{0}\left(x\right)}{c_{0}\left(x\right)}=-2\cdot \frac{C_{13}\left(x\right)}{C_{33}\left(x\right)}\cdot \frac{a\left(x\right)-a_{0}\left(x\right)}{a_{0}\left(x\right)}$$

where *a*(*x*) and *c*(*x*) represent the measured in-plane and out-of-plane lattice constants of the Al_x_Ga_1−x_N layer, *a*_0_(*x*) and *c*_0_(*x*) represent the in-plane and out-of-plane lattice constants of the free-strained Al_x_Ga_1−x_N layer, and *C*_13_(*x*) and *C*_33_(*x*) are the elastic constants of the Al_x_Ga_1−x_N layer. The lattice constants of free-strained Al_x_Ga_1−x_N were calculated using Vegard’s law and the following Equation (4) [32]:

(4) $$\{\begin{array}{c} a_{0}\left(x\right)=a_{0}^{AlN}\cdot x+a_{0}^{GaN}\cdot \left(1-x\right)\\ c_{0}\left(x\right)=c_{0}^{AlN}\cdot x+c_{0}^{GaN}\cdot \left(1-x\right) \end{array}$$

where $a_{0}^{AlN}$, $c_{0}^{AlN}$, $a_{0}^{GaN}$, and $c_{0}^{GaN}$ are the lattice constants of strain-free AlN and GaN. By employing the linear interpolation method given in Equation (5):

(5) $$\{\begin{array}{c} C_{13}\left(x\right)=C_{13}^{AlN}\cdot x+C_{13}^{GaN}\cdot \left(1-x\right)\\ C_{33}\left(x\right)=C_{33}^{AlN}\cdot x+C_{33}^{GaN}\cdot \left(1-x\right) \end{array}$$

where the elastic constants of AlN and GaN are denoted by $C_{13}^{AlN}$, $C_{33}^{AlN}$, $C_{13}^{GaN}$, and $C_{33}^{GaN}$, which were taken as 108 GPa, 373 GPa, 103 GPa, and 405 GPa [33], respectively. Then, the elastic constants *C*_13_(*x*) and *C*_33_(*x*) of the Al_x_Ga_1−x_N layers were determined using the above equations.

From Equations (3)–(5), a cubic equation about the Al content x was obtained. Jointly solving the univariate cubic equation of the above composition, the Al content x and the strain relaxation ratio R of the AlGaN epilayer of samples HVPE-AlN and 1700 °C annealed HVPE-AlN were determined to be 70.29% and 0.13, and 71.69% and 0.21, respectively, which quantified the strain states.

### 3.7. Electroluminescence Spectroscopy (EL) Study 

It has been proved that [34] when the strain relaxation ratio of AlGaN material is less than 36%, the PL luminescence intensity of AlGaN quantum increases well with the increase in the strain relaxation ratio. According to the theory of non-radiative recombination, strain relaxation can lower the vacancy concentration as a result of the reduced formation energy due to the partial alleviation of the compressive strain in AlGaN.

The WPE corresponds to the ratio of the optical output power *P*_out_ and the electric input power, that is, the product of the operating current *I*_lop_ and drive voltage *V*. Therefore, the following Equations (6) and (7) were obtained [35]:

(6) $$\mathrm{WPE}=\frac{P_{\mathrm{out}}}{I_{\mathrm{lop}}\times V}$$

(7) $$\eta_{\mathrm{EQE}}=\eta_{\mathrm{IQE}}\times \eta_{\mathrm{extr}}$$

where the light extraction efficiency *η*_extr_ quantifies the UV photon escape probability from the semiconductor chip. 

**Figure 6:**
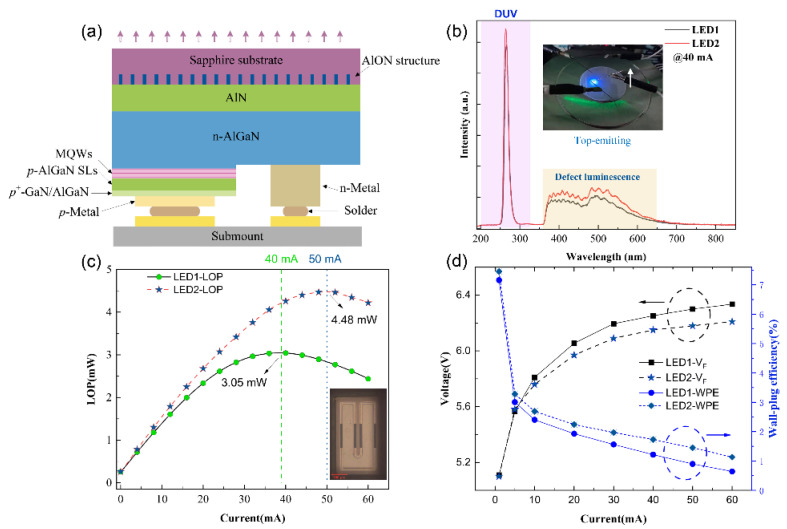
(**a**) Structure diagram of a 265 nm LED2 flip-chip structure; (**b**) EL spectrum of an epitaxial wafer at 40 mA; (**c**) light-emitting surface of a 265 nm LED chip; (**d**) electrical performance test results of LED1 and LED2.

To study the gap between the performance of the DUV-LED based on the HVPE-AlN HTA template and traditional devices, we selected unannealed HVPE-AlN and AlN annealed at 1700 °C to fabricate LED1 and LED2, respectively. Compared with LED1, in addition to the improvement in epitaxial material, the LED2 structure contained many rectangular AlON structures in the sapphire substrate near the AlN side, as shown in Figure 6a; they cannot be ignored. In Figure 6b, we tested the electroluminescence (EL) spectra of the epitaxial wafers of the two groups of samples at 40 mA. In the spectra, the luminous intensity of the LED2 sample was slightly (approximately 10%) higher than that of LED1. Epitaxial AlGaN based on HTA-AlN will reduce the dislocation density in AlGaN [36]. The reduction in the dislocation density is very important for the realization of a high-LOP DUV-LED [37]. The lower dislocation density in the AlGaN layer enables a higher carrier transfer efficiency and, thus, higher LOP.

The yellow band luminescence peak of the LED2 epitaxial wafer was stronger, which may have been due to the incorporation of more C in the process of the epitaxial formation of the smaller compressive strain AlGaN material after HTA [38]. Figure 6d shows that LED2 based on AlN after HTA had a lower working voltage (VF) than LED1, which may have been due to the higher quality of the epitaxial material of the LED2 sample and the lower concentration of impurity defects in the crystal. The LOP of LED2 reached a maximum of 4.48 mW at 50 mA, which was approximately 57% higher than that of LED1. 

The epitaxial chip structure and fabrication process of the two LEDs samples are exactly the same. Compared with flip-chip LED1, the LOP of flip-chip LED2 with light from the sapphire substrate side is increased by 38% at 40mA. This value is much larger than the EL test result of light emitted from the electrode side. The total internal reflection (TIR) of light at certain angles of incidence on the interface between two lossless dielectric media is a well-known aspect of Fresnel reflection [39]. In the case of the flip-chip module, light with incident angles larger than 34.6° for DUV LED is trapped inside the sapphire slab [40]. Fresnel reflections at the sapphire/air interface are another source of optical loss. At the air/sapphire interface, the introduction of a material with an intermediate refractive index (RI) between air and sapphire can lessen the Fresnel reflection. The RI of porous Al_2_O_3_ can be tuned from 1.82 to 1. Double-layer anti-reflection (AR) coating made of porous Al_2_O_3_ with different RI can improve *η*_extr_ by 8% for DUV LED with an emission wavelength of 265 nm [41].

Thus, Fresnel reflection can be reduced with the incorporation of a material having an intermediate refractive index (RI) of AlN and sapphire at the AlN/sapphire interface. The RI of α-Al_2_O_3_ is 1.66–1.61 in the 240–840 nm wavelength interval [42]. The RI of AlN is 2.2–2.4 in the 250–300 nm wavelength interval [43]. The RI of Al_5_O_6_N is 1.77–1.91 in the 240–600 nm wavelength interval [44]. Therefore, the appearance of AlON suppresses the Fresnel reflection at the AlN/sapphire interface, improving the *η*_extr_ of DUV-LED devices.

### 3.8. Microscopic Light Distribution Study 

The brightness and color of the light source surface were not uniformly distributed. Various factors will cause changes in the uniformity of light emitted from the LED surface, such as volcanic defects introduced during AlGaN epitaxial growth [45], electrode patterns [46], uniform carrier distribution [47], and modified reflective n-type electrodes [48]. The surface luminous uniformity of LED2 is slightly lower than that of LED1. This has been caused by the fact that the AlON structure on the AlN/sapphire interface was not distributed in a uniform manner. In the following research, methods modified by reflective n-type electrodes can be used to improve the luminous uniformity of LED2 containing the AlON structure.

**Figure 7:**
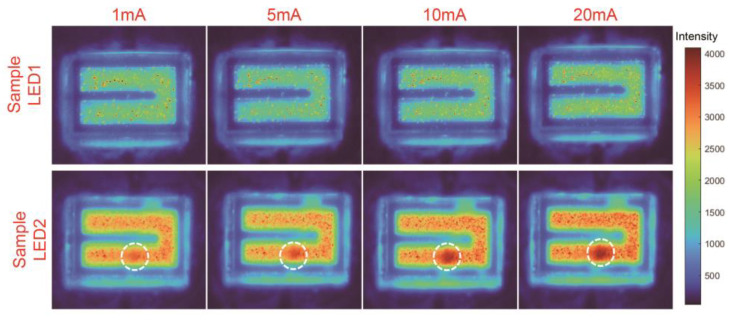
The two LED chips were tested for microscopic light distribution at 1 mA, 5 mA, 10 mA, and 20 mA.

The traditional light source test method did not accurately describe the spatial light distribution characteristics of the light source surface, and it was prone to uneven chromaticity and brightness. Therefore, it was necessary to use the microscopic light distribution test to evaluate the luminous uniformity of the die. The test spectral range was 200–280 nm, and the light intensity distribution matrix obtained by the test was plotted on the same intensity axis through MATLAB software. From Figure 7, we observed that the luminous intensity of LED2 was always significantly higher than that of LED1 with increasing injection current. However, when the current exceeded 10 mA, the light intensity of the dotted line marking area of LED2 was significantly higher than that of other areas of LED2. This may have been because the AlON structure was not uniformly distributed on the substrate side and the AlON structure had a higher structure density in the red region. Therefore, the appearance of AlON suppresses the TIR by reducing the Fresnel reflection at the LED2 dotted line marking area, improving the LOP of the DUV-LED die.

## 4. Conclusions

The surface roughness of the HVPE-AlN samples decreased, and the crystal quality increased, with the increasing annealing temperature. Volcano-like protrusions on the surfaces of the HVPE-AlN samples after annealing at temperatures above 1700 °C were mainly due to stacking faults in the (1014)slip plane. The 265 nm flip-chip structure LED die based on HVPE-AlN samples after high-temperature annealing had higher WPEs than the flip-chip structure LED based on unannealed AlN. The regular AlON structure at the interface after high-temperature annealing may improve the light extraction efficiency of flip-chip deep ultraviolet devices; this structure has value for use in research to improve the light extraction efficiency.

## Figures and Tables

**Table 1:** Stress modes in four groups of AlN samples.

Sample	*ω* (cm^−1^)	*ω*_0_ (cm^−1^)	Δ*ω* (cm^−1^)	$\sigma_{\mathrm{xx}}(GPa)$	Mode	Type of Strain
Strain-free AlN	657.40	657.40	0	0	AlN-E_2_(high)	Unstrained
HVPE-AlN	655.31	657.40	−2.09	−0.82	AlN-E_2_(high)	Tensile stress
Annealed 1500 °C	656.49	657.40	−0.91	−0.35	AlN-E_2_(high)	Tensile stress
Annealed 1600 °C	656.63	657.40	−0.77	−0.31	AlN-E_2_(high)	Tensile stress
Annealed 1700 °C	657.08	657.40	−0.32	−0.13	AlN-E_2_(high)	Tensile stress

## Data Availability

All data that support the findings of this study are included within the article.
